# Family Functioning and Psychological Health of Children with Mentally Ill Parents

**DOI:** 10.3390/ijerph16071278

**Published:** 2019-04-10

**Authors:** Silke Wiegand-Grefe, Marlit Sell, Bonnie Filter, Angela Plass-Christl

**Affiliations:** Clinic for Child- and Adolescent Psychiatry, Psychotherapy and Psychosomatic, University Medical Centre Hamburg-Eppendorf, Martinistr. 52, 20246 Hamburg, Germany; s.wiegand-grefe@uke.de (S.W.-G.); m.sell@uke.de (M.S.); b.filter@uke.de (B.F.)

**Keywords:** family functioning, psychological health, children of mentally ill parents, parental mental disorder

## Abstract

Parental mental illness can be linked to reduced family functioning, which is associated with more conflicts, less adaptability and cohesion as well as a disorganized pattern of everyday planning. Concurrently, family functioning is an important moderator for the influence of parental mental disorders on the development of the children. Consequently, the current study addresses the correlation of family functioning in families with mentally ill parents and the psychological health of the children. The sample consists of 67 mentally ill parents. Both parents and therapists completed questionnaires related to family functioning and the psychological health of the children. Family functioning was rated as dysfunctional in 38% of the families. The psychological health of the children was classified as clinical or subclinical in 43% of the cases. 52% of the children were rated to have no psychological problems. In families with good family functioning, children were assessed to have less psychological problems than in families with poor functioning. Children outside the clinical range lived in families with good family functioning and vice versa. Significant positive correlations were found between the FB-A scales, the CBCL/4-18 syndrome scales and the CBCL/4–18 total score. Results indicate that family functioning and psychological health of children in families with mentally ill parents correlate closely and represent potential targets for future family interventions.

## 1. Introduction

Parental mental illness can be associated with reduced family functioning. The majority of studies related to parental mental disorders examine family functioning in families that deal with parental depression [1,2]. Keitner and Miller [3] found worse family functioning in families with a parent suffering from depression not only in comparison to a non-clinical control group but also compared to families dealing with other parental mental disorders. In this study, reduced family functioning was associated with more conflicts, less adaptability and cohesion, as well as a disorganized pattern of everyday planning and assignment of chores. Concordantly, in a naturalistic study, Slatcher and Trentacosta [4] demonstrated an association between parental depressive symptoms and the problem behavior of their children in daily life. 

In families with major depression and bipolar I disorder, Weinstock and colleagues [5] discovered a significant family dysfunction that persisted during the depressive episode and the non-depressive interval. Neither were correlations found between the type of episode (depressive or manic) and family functioning nor could family functioning predict a future episode [6]. 

Boegels and Brechman-Toussaint [7] identified a higher percentage of dysfunctional family structures in families with parental anxiety disorders in comparison to psychologically healthy controls or other parental disorders. 

Maternal borderline personality disorder is also found to have an effect on family life [8]. The insensitive behavior of borderline mothers tends to increase children’s distress. In their comprehensive review including 33 studies, Eyden [9] and colleagues state that mothers with borderline personality disorder were more likely to engage in maladaptive interactions with their offspring characterized by insensitive, overprotective, and hostile parenting compared to mothers without borderline personality disorder. The results suggest that vulnerability from mothers to offspring may be partly transmitted via maladaptive parenting and maternal emotional dysfunction. 

In families with various parental psychiatric diagnoses, family functioning was altogether worse compared to healthy control families, e.g. [10,11,12]. Specific differences between the particular psychiatric diagnoses regarding the dimensions of family functioning were not found. In contrast, family functioning was strongly associated with the severity of the parental illness. Altogether, the results indicate that parental mental disorders constitute an important risk factor for family functioning.

Family processes do not only play an important role in the course of the parental illness, they also influence the children’s development to a great extent. Family functioning was identified to be an important moderator for the influence of parental mental disorders on the development of the children. Hammen, Brennan and Shih [13] described a significant link between family functioning and the prevalence of depression in children with depressive mothers. In some studies, disadvantageous family interactions prove to have more impact on child development than the parental depression itself [14,15,16,17,18]. Warner and colleagues [19] specified that chaotic family conditions serve as an independent predictor for the children’s risk to develop an affective mental illness or a panic disorder, when the parents suffer from the respective disorders. 

In families with depressive parents, an indirect correlation between parental depression and child bipolar disorder was discovered, which was mediated by poor family functioning and frequent familial conflict. However, the indirect correlation of familial processes with the bipolar disorder of the children was smaller than the direct correlation with the bipolar disorder of the parents [20].

In the offspring of parents with bipolar disorder low levels of parental structure had robust effects on emotional and behavioural problems in middle childhood, while levels of parental control emerged as strongest mediator of the relation between parent´s bipolar disorder and offspring psychopathology in puberty [21]. 

Family functioning moderated the impact of interparental violence, child abuse and other traumatic experiences on child post-traumatic symptoms [22].

In a longitudinal study over 4 years with 199 three-year-old children with behaviour problems, Harvey and colleagues [23] demonstrated that children with behaviour problems who were exposed to overreactive parenting practices, maternal depression, marital conflict, and lower family income tended to have more oppositional defiant disorder (ODD) symptoms 3 years later. Children who met the criteria for attention-deficit/hyperactivity disorder at 6 years were less likely to show improvement in ODD symptoms from 3 to 6 years of age and they were more likely to experience (?) negative parenting practices, marital conflict, and parental depression during the preschool years. The authors stress the importance of early family functioning in the development of ODD. 

A longitudinal adoption study with children of schizophrenic mothers showed that children’s risk of developing a psychiatric disorder increased only when the children were raised in a dysfunctional family environment [24]. 

Apart from these diagnosis-specific findings, a psychiatric disease of a parent in general is an important factor for the correlation of family functioning with the adaptation in childhood [25] or internalizing problems in adolescence [26]. 

On the one hand, a parental mental illness is associated with worse family functioning independently of the kind of the parental diagnosis and more contingent upon the severity of the illness. On the other hand, worse family functioning is associated with behavioral and psychological problems of the children. Consequently, the present study investigates the association of family functioning and psychological health of children in families with mentally ill parents. It is hypothesized that children of mentally ill parents in functional families exhibit less psychological problems than children in dysfunctional families. 

## 2. Materials and Methods

### 2.1. Procedure

The study was conducted at the University Medical Centre Hamburg-Eppendorf, Germany. Within a 9-month period, all in-patients referred to the Clinic of Psychiatry and Psychotherapy were registered. Inclusion criteria were: patient aged 18–60 years, parent of at least one minor child between 0–18 years, staying at least one week in the hospital, having sufficient knowledge of German language and giving informed consent for the participation in the study. Exclusion criteria were: previous participation in the study in case of repeated hospitalization and severe psychiatric or cognitive impairments. Family functioning and psychological health of the children were assessed by parents answering standardized questionnaires. 

### 2.2. Participants

Overall, 964 patients were registered in the study. Among this sample, 558 patients had no children (58%), 104 patients had grown-up children above 18 years (11%) and 135 patients were older than 60 years (14%). Of the remaining 167 parents, 42 (25%) were not willing to participate in the study and further 39 parents (23%) did not fulfill the inclusion criteria. N = 86 parents participated in the study. For data analysis, the answers of 67 parents could be used. Nineteen patients had to be excluded due to missing data or they did not have continuous contact with their children. All participants provided informed consent and the study was approved by the local research and ethic committee of the Aerztekammer Hamburg.

The sample consisted of 67 mentally ill parents including 33 fathers and 34 mothers, each sex representing about 50% of the sample. The age ranged from 22 to 58 years (M = 41.10, SD = 7.27). 52% of the parents were married, 19% were divorced, 3% lived separated from their spouse and 22% were single. One patient was widowed (1.5%) and one patient (1.5%) did not specify his marital status. The parents’ school leaving certificate was senior high-school (43%), graduation from intermediate secondary school (31%) and secondary general school (20%). One patient each (1.5%) was without school degree, or had another school leaving certificate. 

Regarding the qualifications, vocational trainings (39%) and University degrees (25%) were the most prevalent qualifications, 7.5% had a polytechnic degree, 7% hold another qualification. Only 15% of the parents had no further qualification, 4% (3 parents) were students or trainees and 3% (2 parents) did not specify their qualification. 36% of the parents were white-collar employees, blue-collar workers, or state employees. 

The parents were diagnosed with the following psychiatric disorders: 23 parents (34%) with mood disorders (ICD-10 F3), 18 parents (27%) with neurotic, stress related and somatoform disorders (ICD-10 F4), 14 (21%) with disorders due to substance use (ICD-10 F1), 11 (16%) with schizophrenia, schizotypal and delusional disorders (ICD-10 F2) and one (2%) with personality disorder (ICD-10 F6). The mean illness duration was 8.3 years (SD = 8.0; range <1 to 34). 

The children were 28 boys (45%) and 34 girls (55%), their age ranged from 4 to 18 years (M = 11.24 years, SD = 4.49). 42 (63%) of the parents stated that their children were living in their household. The remaining 25 children (37%) were living separated from the parent in question. All parents in the study had continuous contact (at least once a week) to their children. Of these 25 children, 18 (72%) lived in the household of the other, non-mentally ill parent. One child lived with relatives or in a foster family. Two children (8%) had an apartment on their own and three children lived in institutional care.

### 2.3. Instruments

Family functioning is a multidimensional construct, which describes the familial organisation and problem solving of the family [27]. In the present study, family functioning was assessed with the Family Sheets (Familienboegen, FB, [27]), an operationalisation of the family model of Cierpka [28] that is based on the “McMaster Model of Family Functioning” [29] and the “Process Model of Family Functioning” [30]. The questionnaire consists of three modules with 28 items each. For the present study, the perspective of the total family (FB-A, Allgemeiner Familienbogen) was considered, a self-report form that respondents rate in terms of how well each item reflects the family’s functioning. The items are rated on a 4-point-Likert-scale and range from “completely true” = 0 to “not true at all” = 3. 

Higher scores reflect greater dysfunction, and T-scores above 60 reflect difficulties in family functioning, T-scores below 50 can be interpreted as strengths in family functioning. The FB-A comprises seven subscales corresponding to the underlying family model. Examples for items of the different scales: Scale 1: Task Fulfillment, Ex: “Responsibilities in the family are fairly distributed”. Scale 2: Role Behaviour, Ex: “We agree as to who has to do what in the family”. Scale 3: Communication, Ex: “We take time to listen to each other”. Scale 4: Emotionality, Ex: “When we get upset in our family, we need long time to get over this”. Scale 5: Affectivity of Relations, Ex: “We are closely united”. Scale 6: Control, Ex: “In our family it is difficult to follow one´s own way”. Scale 7: Values and Norms, Ex: “We have the same opinions about what is right and wrong”. The subscales can be summed up into a total score. The FB-A has been standardized with healthy families. The intercorrelation of the seven scales varies between r = 0.28 and r = 0.63. All scales load on a common factor with an explanation of variance of 52%. The internal consistency measured by Cronbach’s Alpha lies between α = 0.46 und α = 0.80 (with α > 0.60 for the most subscales). The 28 items of the FB-A show a Cronbach’s Alpha of α = 0.88. 

In addition, family functioning was assessed by the attending psychiatrists with the “Global Assessment of Relational Functioning Scale“(GARF, [31]), a DSM-IV measure of adaptive functioning. The scale is based on the dimensions problem solving, organization and emotional climate. The one-item measurement that characterizes the overall functioning of a family is rated on a 100-point scale, that is divided into five areas of functioning, higher scores representing better family functioning. A score between 1–20 indicates that the relational system is “too dysfunctional to retain a continuity of contact and attachment”, a score of 21–40 indicates that the relational system is so impaired that “periods of satisfactory relating are rare”. A range between 41–60 points displays that there are times of satisfying relationships but that “unsatisfying relationships tend to predominate”, 61 up to 81 points indicate that most difficulties in the relationships can be resolved, whereas a score from 81 up to 100 suggests that the relationships function in a satisfactory manner. 

For the assessment of the psychological health of the children, parents were administered the German version [32] of the Child- Behavior-Checklist (CBCL/4-18, [33]), that assesses behavioural and emotional problems in children and adolescents. Parents rate their children on 113 items using a 3-point (Likert-) scale (0 = “not true”, 1 = “somewhat or sometimes true”, 2 = “often true”). A total problem score, two broadband scores (Internalizing and Externalizing Problems) and eight different syndrome scales were generated. The good reliability and validity established for the original CBCL-version [33] have been confirmed for the German edition used in this study [32]. The German CBCL encompasses children/youths aged 4–18 in accordance with the US 1991 CBCL-version. 

### 2.4. Data Analysis

All statistical analyses were conducted using the software package IBM SPSS (IBM, Armonk, NY, USA). For standardised instruments, missing values were replaced according to the guidelines of the respective manuals. Values of the FB-A and the CBCL-4/18 were transformed to T-values. Comparisons of mean values were performed by t-tests for independent samples. Correlations were calculated according to Spearman and Pearson. The general level of significance was defined at 5%, this is indicated as significant. A level of significance of 1% is indicated as very significant and a level of significance of 0.1% as highly significant. Correlations are indicated as low at 0.10, as medium at 0.30 and as high at 0.50. 

## 3. Results

### 3.1. Family Functioning from the Parents and Therapists Perspective

Overall, the dimensions of family functioning ranged in the lower average, according to the self-assessment of the mentally ill parents (Table 1). In five scales (namely task fulfillment, communication, emotionality, control and the total score), the mean values of the T-scores were situated in the lower average, slightly under 60. None of the scales’ scores was under a T-score of 50 that would imply good family functioning. The scales “role behavior” and “values and norms”, were rated to be areas of rather good functioning.

Regarding the distribution of T-scores, a considerable percentage of families, namely 38%, ranged within the area of dysfunction (T-score above 60) across all scales. On five dimensions (task fulfillment, communication, emotionality, affectivity of relations and control), another 22% of the families displayed a T-score three standard deviations above the mean (T-Score > 70). Thus, although the average T-score was in the normal range, a considerable percentage of patients described their family’s functioning as problematic. On the other hand, 28–42% of the families (35% across the scales) were within a range of good functioning (T-score below 50). Almost no patient though, described very good functioning with a T-score below 30. We also examined the distribution of the categorized T-scores on the total score that could be calculated for N = 69 patients. No family ranged beneath a T-score of 30 that would indicate very good family functioning. Three families (4%) were within the range of 30–39, 21 families (31%) between 40–49, 18 families (27%) between 50–59, 12 families (18%) between 60–69, and 13 families (19%) had a T-score above 70. It is striking that considerably more families were assessed on the dysfunctional extreme than on the functional end of the spectrum. 

In the therapists’ ratings (GARF, N = 67), the majority of families were described to exhibit dysfunctional relationships. Only one family was rated to display satisfactory functioning (score 81–100). Whereas 18 families (27%) exhibited some difficulties over time (score 61–80) or only occasional good functioning (41–60), 14 families (21%) were assessed to be seriously dysfunctional (score 21–40). 10 families (15%) were rated to be too dysfunctional to maintain contact (score 1–20). Regarding the interrater-agreement a significant average association (r = −0.39, *p* = 0.003) can be stated. 

### 3.2. Psychological Health of the Children from the Parents Perspective

The T-scores of the two broadband scales and the total problem score showed mean values under 60, thus lying in the normal range (T < 59). More than half of the parents rated their children as having no internalising (54% of the parents) or externalising (52% of the parents) problems. At the same time, more than one third of the parents rated their children to be in the clinical or subclinical range. On the total problem score, 43% of the children were assessed in the clinical/subclinical range (Table 2).

### 3.3. Family Functioning and Psychological Health of the Children

With t-tests for independent samples, families with good family functioning (FBA total-score > 60) were compared to families with poor family functioning (FBA total-score < 60) regarding their children´s psychological problems measured with the CBCL/4-18 total score, internalizing and externalizing problems. The test revealed that families with good versus poor family functioning differed significantly regarding the psychological health of the children on the total score and the two broadband scales of the CBCL/4-18 (Table 3). In families with good functioning, children were rated to have less problems than children in families with poor functioning. 

In a further step, families were differentiated into two groups by a cut-off of 63 in the CBCL/4-18 of their children as a T value of 63 and higher represents clinically relevant behavioural and psychological problems (Table 4). 

Using t-Tests for independent samples we tested for significant differences regarding family functioning in the two groups. In the group with children in the clinical range, the total score for family functioning was significantly higher—indicating worse family functioning—compared to children in the non-clinical range. Thus, children who were rated to be not at risk for a mental disorder lived in families exhibiting good family functioning and vice versa.

Further T-Tests for independent samples for each of the seven dimensions of the FB-A confirmed this result partially. In the total score and the two broadband scales of the CBCL/4-18 significant differences between children from functional vs. dysfunctional families were detected regarding the FB-A scales “communication”, “emotionality” and “values and norms”. Regarding the FB-A scales “role behaviour” and “affectivity of relations”, significant differences were determined for the total score and internalising problems of the CBCL/4-18. Only the scales “role behaviour” and “task fulfilment” did not differentiate significantly between children from functional vs. dysfunctional families. 

Using correlation analysis, the detected differences were examined for relations. Positive (linear) correlations between the scales of the CBCL/4-18 and of the FB-A were hypothesised (Table 5). Significant correlations were found for the FB-A scales “role behaviour”, “affectivity of relations”, and “psychological problems” of the children measured by the CBCL/4-18. 

The least significant correlation was r = 0.241 (*) (for the scales “somatic complaints” and “role behaviour”), the highest was r = 0.429 (**) (for the scales “aggressive behaviour” and “values and norms”). Small and non-significant correlations were only found regarding the FB-A scale “control”. Most correlations of the FB-A scales were found with the CBCL/4-18 scale “aggressive behaviour”. With the exception of the FB-A scale “control”, all FB-A scales correlated significantly with the scale “aggressive behaviour”.

Most correlations of the CBCL/4-18 scales were found with the FB-A scale “role behaviour”: With the exception of the CBCL/4-18 scale “delinquent behaviour”, all CBCL/4-18 scales correlated significantly with the scale “role behaviour”. Many correlations with the CBCL scales were also found for the scales “affectivity of relations” and “values and norms”. Contrary to expectations, the scale “communication”, which is very important for family life, did correlate only with two CBCL/4-18 scales, namely “thought problems” and “aggressive behaviour”. However, two other scales, which are important for family interaction, that is “emotionality” and “affectivity of relations”, showed six partially significant and eight significant or very significant correlations.

## 4. Discussion

In the present study, more than one third (38%) of the mentally ill parents described their families as dysfunctional. This proportion of dysfunctional families is higher than in the normal population and comparable to other samples of mentally ill patients [11,12,34].

The prevalence of children’s emotional and behavioural problems in the present study assessed by their mentally ill parents was 43%. This rate clearly exceeds the prevalence in the general German population where a recent meta-analysis of 33 epidemiological studies estimated a prevalence of 17.6% [35]. This percentage was confirmed in a recent epidemiological study, where 17.2% of the children and adolescents reported mental health problems [36]. Our findings correspond to previous results, implicating that the prevalence of mental health problems among children of mentally ill parents is 2 to 5 times higher than in the general population [37,38]. In a similar survey [39], where mentally-ill parents assessed their children’s mental health with the SDQ (Strengths and Difficulties Questionnaire; [40]), 35% of the children scored in the clinical range and 12% in the subclinical range. Overall, 47% of the children exhibited clinical/subclinical symptoms – a similar but slightly higher rate compared to the present findings. 

In our sample of mentally ill parents, higher levels of family dysfunction were associated with more child psychological problems and vice versa. An association of family functioning with children’s psychopathology was also found in previous studies that investigated family factors for the outcome of paediatric obsessive-compulsive disorder [41] and the role of family experiences in the early development of oppositional defiant disorder [23]. Our results confirm the findings of these studies in a sample of mentally ill parents.

Correlation analysis revealed significant correlations between child psychological problems and the dimensions of family functioning. However, as all significant correlations were in the mean range, the results have to be interpreted with caution. A high intercorrelation of the FB-A scales is another reason for a limited interpretability. Most correlations of the FB-A scales were detected for the CBCL/4-18 scale “aggressive behaviour”. These results indicate that the parents in our sample associate aggressive behaviour in their children with a dysfunctional family climate. High associations with the family climate were also found for “social withdrawal”, “attention problems”, “internalizing problems” and the “total score”. With the CBCL scales “somatic complaints”, “social problems” and “thought problems” only few correlations were significant. Thus, parents did not estimate a strong association of these symptoms of their children with the family climate. 

Our results regarding correlations of the CBCL/4-18 scales with the FB-A scales largely confirm the findings of Friedman and colleagues [12]. In their study, the FB-A scale “control” was also the only scale that did not correlate with the psychological health.

## 5. Conclusions

The data presented in this study offers some intriguing information about the association of family functioning and psychological problems of children in families with mentally ill parents. In a recent study [42] with children suffering from obsessive-compulsive disorder, it has been demonstrated, that targeting family functioning constitutes an important and successful treatment mechanism. A recent evaluation of a family-centred preventive intervention for military families [43] revealed improvement of psychological health outcomes in parents and improvement over time in emotional and behavioural symptoms and in prosocial behaviour in children combined with the reduction of unhealthy family functioning. 

As family relations play an exceedingly important role in the transmission of mental illness [44,45], family functioning constitutes a relevant target for interventions in families with mentally ill parents. Preventive interventions for families with mentally ill parents address e.g., parent-child interactions [46] and report improvements in this respect [47]. First results show promising improvements in parenting and child behaviour outcomes in a web-based parenting intervention [48]. When conceptualizing interventions for families with mentally ill parents, these associations should consequently be considered [49].

As to the question, whether preventive interventions for families with mentally ill parents are effective recent meta-analyses revealed, that preventive interventions decreased the risk for mental illness in the children by 40% and reduced symptom scores in the offspring [50]. Mother-infant interactions as well as mothers´ and children´s behaviour and children´s psychopathology improved consistently with preventive interventions. Overall, interventions addressing parents and children jointly produced larger effects [51]. Therefore, preventive interventions addressing parents and children, family functioning and social support seem to be best suitable to prevent transmission of mental illness. 

### Limitations and Future Lines

As the data used in the present study are cross-sectional, no causal inferences can be made. The information about the psychological problems of the children was assessed by asking the mentally ill parent. We chose this perspective because, from our experience, mentally ill parents often express concerns regarding the scientific assessment of their children. Thus, results could be biased by a distorted realty perception and restricted power of judgement, which often accompany psychiatric diseases. The assessment may also be constricted because not all the parents lived together with their children, though a continuous contact between parents and children was an inclusion criterion. Social desirability may also influence the results. The small sample (N = 67) restricts interpretation of the group comparisons. 

Future research should employ longitudinal designs to further elaborate the relationship between family functioning and psychological problems of children in families with mentally ill parents. In addition, future research should consider diverse perspectives (e.g., patient, partner and children) to gain information about different perceptions of family functioning and psychological problems of the children in the family.

## Figures and Tables

**Table 1:** Values of the FB-A.

	Scale 1: Task Fulfillment	Scale 2: Role Behaviour	Scale 3: Communication	Scale 4: Emotionality	Scale 5: Affectivity of Relations	Scale 6: Control	Scale 7: Values and Norms	Total Score
Valid	67	67	66	67	67	67	67	67
Missing	0	0	1	0	0	0	0	0
Mean	58.54	54.12	59.17	58.38	59.9	58.34	56.16	59.54
Median	56.68	54.44	52.81	58.57	53.51	56.39	55.89	56.09
Standard-deviation	14.95	11.45	18.54	14.87	18.84	16.14	13.82	17.37
Minimum	30.62	27.30	33.11	35.97	36.16	32.05	33.14	34.53
Maximum	89.98	76.71	110.08	100.34	100.82	95.17	96.07	109.11
Percentile								
25%	47.64	46.31	45.65	47.90	45.09	46.65	44.74	46.14
50%	56.68	54.44	52.81	58.57	53.51	56.39	55.89	56.09
75%	69.79	62.37	74.26	69.25	76.05	69.06	64.14	72.47

**Table 2:** T-values of the CBCL/4-18 in the normal, subclinical and clinical range.

CBCL/4-18	Range	Frequency	Percentage (rounded)
Internalizing Problems	Normal Range	36	54
	Subclinical Range	5	7
	Clinical Range	18	27
	Total	59	88
	Missing	8	12
Externalizing Problems	Normal Range	35	52
	Subclinical Range	10	15
	Clinical Range	14	21
	Total	59	88
	Missing	8	12
Total Score	Normal Range	35	52
	Subclinical Range	9	13
	Clinical Range	20	30
	Total	64	95
	Missing	3	4

**Table 3:** T-Test for independent samples for functional vs. dysfunctional families regarding psychological problems of the children.

CBCL/4-18 Scales	T-Value FB-A Total Score	N	M	SD	t	df	*p* (2-tailed)
T-Value Total Score	≥60 <60	28 36	63.07 54.88	13.2 7.85	2.90	41.3	0.006
T-Value Internalizing Problems	≥60 <60	27 32	61.88 53.09	12.8 8.93	3.17	57	0.002
T-Value externalizing Problems	≥60 <60	27 32	59.85 54.31	11.9 8.94	2.06	57	0.044

Note: N = Sample size; M = Mean value; SD = Standard deviation; t = Test statistic for comparison of means; *p* (2-tailed) = two-tailed level of significance.

**Table 4:** T-Test for independent samples for children with vs. without psychological problems regarding family functioning of their families.

Family Functioning	T-Value CBCL Total Score	N	M	SD	t	df	*p* (2-tailed)
FB-A-Total Score	≥63 <63	22 42	67.68 55.36	18.21 15.93	2.797	62	0.007

*Note*: N = Sample size; M = Mean value; SD = Standard deviation; t = Test statistic for comparison of means; *p* (2-tailed) = two-tailed level of significance.

**Table 5:** Correlations of psychological problems of the children and family functioning. (Correlation analysis of CBCL/4-18 and FB-A Scales, Spearman´s rank correlation coefficient).

CBCL/4-18 Scales	Scale 1: Task Fulfillment	Scale 2: Role Behaviour	Scale 3: Communication	Scale 4: Emotionality	Scale 5: Affectivity of Relations	Scale 6: Control	Scale 7: Values and Norms
Social Withdrawal	0.355 (**)	0.305 (*)	0.238	0.349 (**)	0.361 (**)	0.195	0.365 (**)
Somatic Complaints	0.145	0.241 (*)	0.123	0.131	0.157	0.045	0.169
Anxious/Depressed	0.181	0.331 (**)	0.212	0.192	0.252 (*)	0.209	0.292 (*)
Delinquent Behavior	0.190	0.268 (*)	0.068	0.187	0.286 (*)	0.133	0.332 (**)
Aggressive Behavior	0.244 (*)	0.275 (*)	0.281 (*)	0.333 (**)	0.274 (*)	0.156	0.429 (**)
Social Problems	0.070	0.203	0.123	0.181	0.254 (*)	0.135	0.235
Thought Problems	0.107	0.252 (*)	0.268 (*)	0.136	0.189	0.187	0.214
Attention Problems	0.262 (*)	0.348 (**)	0.120	0.277 (*)	0.256 (*)	0.043	0.400 (**)
Internalizing Problems	0.300 (*)	0.398 (**)	0.230	0.263 (*)	0.274 (*)	0.179	0.343 (**)
Externalizing Problems	0.223	0.296 (*)	0.80	0.286 (*)	0.235	0.131	0.331 (*)
Total Score	0.266 (*)	0.384 (**)	0.246	0.307 (*)	0.281 (*)	0.172	0.389 (**)

** The correlation is significant on a 0.01 level (one-tailed); * The correlation is significant on a 0.05 level (one-tailed).
